# Clustered Cases of Waterborne Hepatitis E Virus Infection, France

**DOI:** 10.3390/v15051149

**Published:** 2023-05-11

**Authors:** Sébastien Lhomme, Sébastien Magne, Sylvie Perelle, Emmanuelle Vaissière, Florence Abravanel, Laetitia Trelon, Catherine Hennechart-Collette, Audrey Fraisse, Sandra Martin-Latil, Jacques Izopet, Julie Figoni, Guillaume Spaccaferri

**Affiliations:** 1 Centre National de Référence (CNR) des Virus des Hépatites à Transmission Entériques (Hépatite A et E), Laboratoire de Virologie, CHU Toulouse, 31300 Toulouse, France; abravanel.f@chu-toulouse.fr (F.A.); izopet.j@chu-toulouse.fr (J.I.); 2 Infinity, Université Toulouse, CNRS, Inserm, UPS, 31024 Toulouse, France; 3 Regional Health Agency of Auvergne-Rhône-Alpes, 15000 Aurillac, France; sebastien.magne@ars.sante.fr (S.M.); laetitia.trelon@ars.sante.fr (L.T.); 4 Laboratory for Food Safety, Université Paris-Est, Anses, 94700 Maisons-Alfort, France; sylvie.perelle@anses.fr (S.P.); catherine.hennechart-collette@anses.fr (C.H.-C.); audrey.fraisse@anses.fr (A.F.); sandra.martin-latil@anses.fr (S.M.-L.); 5 Santé Publique France (French National Public Health Agency), 63000 Clermont-Ferrand, France; emmanuelle.vaissiere@santepubliquefrance.fr; 6 Santé Publique France (French National Public Health Agency), 94410 Saint-Maurice, France; julie.figoni@santepubliquefrance.fr

**Keywords:** hepatitis E virus, clustered cases, drinking water, waterborne infection

## Abstract

The identification of seven cases of hepatitis E virus infection in a French rural hamlet in April 2015 led to investigations confirming the clustering and identifying the source of the infection. Laboratories and general practitioners in the area actively searched for other cases based on RT-PCR and serological tests. The environment, including water sources, was also checked for HEV RNA. Phylogenetic analyses were performed to compare HEV sequences. No other cases were found. Six of the seven patients lived in the same hamlet, and the seventh used to visit his family who lived there. All HEV strains were very similar and belonged to the HEV3f subgenotype, confirming the clustering of these cases. All the patients drank water from the public network. A break in the water supply to the hamlet was identified at the time the infection probably occurred; HEV RNA was also detected in a private water source that was connected to the public water network. The water flowing from the taps was quite turbid during the break. The private water supply containing HEV RNA was the likely source of the contamination. Private water supplies not disconnected from the public network are still frequent in rural areas, where they may contribute to public water pollution.

## 1. Introduction

Hepatitis E virus (HEV) is the most common cause of acute viral hepatitis in the world [1]. HEV is a small virus with a positive-sense, single-stranded ~7.2 kb RNA genome that contains three open reading frames (ORF), ORF1, ORF2 and ORF3. ORF1 encodes a non-structural protein about 1693 amino acids (aa) long, with at least four putative functional domains: methyltransferase, cysteine protease, helicase and RNA-dependent RNA polymerase (RdRp). ORF2 encodes the 660 aa capsid protein, which has been divided into three domains: S (shell), M (middle) and P (protruding). It was recently shown that ORF2 also encodes a secreted free form of the capsid protein (ORF2s) that differs from the actual capsid protein, ORF2i (for infectious). Lastly, ORF3 encodes a 113 or 114 aa phosphoprotein, depending on the genotype, that has ion channel activity and is involved in virus egress from infected cells [2].

This small RNA virus belonging to the *Hepeviridae* family has been assigned to subfamily *Orthohepevirinae* for strains infecting mammals and birds and to subfamily *Parahepevirinae* for strains infecting fish [3]. The subfamily *Orthohepevirinae* has four genera including the genera *Paslahepevirus* and *Rocahepevirus* that infect humans, domestic and wild mammals, the genus *Chirohepevirus* that infects bats and the genus *Avihepevirus* that infects birds. Most human infections are due to *Paslahepevirus balayani* genotypes 1, 2, 3 and 4, and less frequently 7. HEV1 and HEV2 are transmitted by the fecal–oral route in countries with suboptimal sanitary conditions, usually via contaminated water. They cause both sporadic infections and occasional large outbreaks [4]. HEV3 and HEV4 are transmitted zoonotically in developed countries from animal reservoirs, such as pigs, wild boar and deer [5]. Most cases are sporadic with unidentified sources of contamination. Large outbreaks are rare, although there have been several well-documented small clusters of cases from a point-source food outbreak [6,7,8]. A waterborne route of HEV transmission is seldom documented in developed countries but it cannot be excluded. HEV3 has been found in untreated and surface water in a number of European, North and South American, and Asian countries [9,10]. Whether the detected RNA is associated with complete infectious particles remains to be determined. A further argument suggesting that water can be implicated in developed countries is that a study of French blood donors found that drinking bottled water was associated with a lower anti-HEV IgG frequency [11]. All the bottled water sold in France comes from underground complex rock systems several hundred meters below the surface, making it likely safe from virological contamination. The HEV found in the blood is lipid-associated without any viral glycoprotein at the surface, which defines a quasi-enveloped virus. Conversely, in feces, and consequently in the environment, HEV is naked because lipids have been removed by the action of bile salts. Naked particles are very resistant in the environment.

The clinical features of hepatitis E are similar to those of acute viral hepatitis caused by other hepatotropic viruses. HEV has a mean incubation period of 40 days [12], and most infections are asymptomatic. Symptoms, when present, are fever, asthenia and nausea, followed by abdominal pain, vomiting, anorexia, and malaise. Jaundice occurs in about 75% of patients. Fulminant hepatitis can occur in pregnant women living in developing countries. The cause of elevated maternal mortality (30%, with most deaths occurring in the third trimester) remains obscure. Immunosuppressed individuals, including solid organ transplant recipients, patients living with the human immunodeficiency virus (HIV), especially those with a T CD4+ count <200/mm^3^ or patients with hematological disease receiving chemotherapy, infected by HEV3 or HEV4 may also develop chronic hepatitis and cirrhosis [12]. Acute HEV infections are diagnosed by detecting serum immunoglobulin M antibodies to hepatitis E (anti-HEV IgM) or by detecting the HEV genome in blood [13]. Reported cases of hepatitis E have increased in industrialized countries due to improved diagnostic tools and screening strategies [13]. For instance, HEV IgG prevalence among blood donors in the Midi-Pyrénées increased from 16.6% to 52.5% when using a more sensitive serological test [14].

A general practitioner in the Auvergne Rhone–Alpes region informed the Regional Health Agency (ARS) about the unusual detection of seven cases of HEV infections in April 2015. All the patients lived in a hamlet of a small village with 200 inhabitants. Symptom onset occurred between March and April 2015. The first case, a 78-year-old man with multiple comorbidities, had died. As a result, anti-HEV IgM and anti HEV IgG serological analyses were prescribed for other families in the hamlet. Six other cases, relatives living in three houses, were then identified. The next step was to describe the outbreak, identify the vehicle and source of contamination, and suggest control measures.

## 2. Materials and Methods

### 2.1. Descriptive Epidemiology

Case definition: We defined a case as everyone living, or having stayed, in the village X, including hamlet Y, who was infected with HEV (detection of anti-HEV IgM or HEV genome) between January and April 2015.

Descriptive analysis and exploration: Cases were actively searched for by local laboratories and general practitioners. We interviewed the cases by telephone using a standardized questionnaire that included sociodemographic characteristics, symptoms and associated medical attention, risk factors such as chronic illness (particularly hepatic), chronic treatment (e.g., corticosteroid), pregnancy, and risk exposure 2–8 weeks before symptom onset (or the date of sampling for the asymptomatic). The risk exposures included contacts with domestic or farm animals, food and water consumption.

### 2.2. Clinical Laboratory Investigations

All samples were stored at −20 °C before analysis. Serological (anti-HEV IgM and IgG) tests were performed on blood sera using Wantai enzyme immunoassays (Wantai Biologic Pharmacy Enterprise, Beijing, China) according to the manufacturer recommendations. The concentrations of HEV RNA in plasma samples were determined by A one-stepRT- qPCR targeting the ORF2/ORF3 overlapping region as previously described [15]. Briefly, HEV RNA was extracted from 140 μL samples (QiaAmp viral RNA minikit; Qiagen, Courtaboeuf, France) according to the manufacturer recommendations. The following primers and probe targeting the ORF2/ORF3 overlapping region were used to amplify a 70 nt fragment: forward primer HEVORF3-S (5′-GGTGGTTTCTGGGGTGAC-3′), reverse primer HEVORF3-AS (5′-AGGGGTTGGTTGGATGAA-3′), and the probe 5′–6-carboxyfluorescein (FAM)–TGATTCTCAGCCCTTCGC–6-carboxytetramethylrhodamine (TAMRA)–3′ [15]. For RT-PCR, the 50 μL reaction mix contained 1 μL of SuperScript III Platinum One-Step quantitative RT-PCR system medium (Invitrogen), 15 μL of RNA, primers (200 nM), probes (150 nM), and 40 U/reaction RNase Out (Invitrogen). Reverse transcription was carried out at 50 °C for 15 min, followed by denaturation at 95 °C for 1 min. DNA was amplified with 50 PCR cycles at 95 °C (20 s) and 58 °C (40 s). The limit of detection was 75 IU/mL. The HEV genotype was determined by sequencing a 348-nucleotide (nt) fragment within the ORF2 gene [16]. For viral load <3 log IU/mL, a successful sequencing is obtained in <50% of attempts.

The neighbor-joining (NJ) tree was constructed with the set of reference sequences proposed by Smith et al. [17,18]. Sequences were aligned using ClustalX2.1 and Treeview 1.6.6 software was used to visualize the tree.

### 2.3. Environmental Investigations

Agents of the Regional Health Agency (ARS) visited village X including hamlet Y on 25 April 2015 in order to determine the geography and public water network architecture. Samples of water were taken from the public network and the hamlet’s private network and sent for analysis at the French Agency for Food, Environmental and Occupational Health & Safety (Anses) [19]. Virus particles were concentrated from 400 mL (well water) and 500 mL (house tap water) samples by membrane filtration under vacuum using a Zetapor (Cuno Filtration SAS 3 M, Cergy Pontoise, France) 47 mm positively charged membrane of pore size 0.45 μm. The filter membranes were then directly incubated for 10 min at room temperature in a 60 mm diameter Petri dish containing 3 mL of NucliSens^®^ easyMAG™ lysis buffer (Biomérieux, Marcy l’Etoile, France).

Nucleic acids were extracted using the NucliSENS^®^ easyMAG™ platform with the “off-board Specific A” protocol according to the manufacturer’s instructions (Biomérieux). Nucleic acids were eluted in 100 µL of elution buffer. An additional purification step was performed with the OneStep™ PCR Inhibitor Removal Kit (Zymo Research, Irvine, CA, USA) to reduce RT-PCR inhibition. A one-step RT-qPCR targeting the ORF2/ORF3 overlapping region was performed. A process control virus (murine norovirus (MNV-1)) and external amplification control RNA (EAC) were applied to monitor the extraction process and to assess any inhibition of amplification. EAC was in vitro transcribed to RNA corresponding to the 5301–5371 positions of genomic sequence AB097812 [19].

The HEV and MNV-1 probes were respectively labeled with the ROX or 6-FAM reporter dyes at the 5′-end, and a HQ2 or BHQ1 at the 3′-end. The sequence of the primer pairs and the TaqMan probes used was as follows: For HEV, the sense primer (HEV-5260-F) was 5′-CGGTGGTTTCTGGGGTGAC-3′, the antisense primer (HEV-5330-R) was 5′-AGGGGTTGGTTGGATGAATATAG-3′ and the TaqMan probe (HEV-5280-T) was 5′-ROX-GGGTTGATTCTCAGC-CCTTCGC–BHQ2-3′. For MNV-1, the sense primer (MNV-3193-F) was 5′-CCGCCATGGTCCTGGAGAATG-3′, the antisense primer (MNV-3308-R) was 5′-GCACAACGGCACTACCAATCTTG-3′ and the TaqMan probe (MNV-3227-T) was 5′-FAM–CGTCGTCGCCTCGGTCCTTGTCAA-BHQ1-3′. All the primers and probes were purchased from Eurofins Genomics (Les Ulis, France) [19]. One-step duplex RT-qPCR amplifications were performed in duplicate on the CFX96™ real-time PCR detection system from Bio-Rad (Marnes-la-Coquette, France).

### 2.4. Ethical Approval

All interviewed participants gave their oral consent. Biological material and clinical data were obtained only for standard viral surveillance (no specific sampling, no modification of the sampling protocol, no questions in addition to the standardized questionnaire). Data were analyzed using an anonymized database. According to the Public Health French law (CSP Art L 1121-1.1), such a protocol does not require written informed consent.

## 3. Results

### 3.1. Descriptive Epidemiology

Seven cases of HEV infection were identified (five men and two women, median age 60 years, range: 27–78), of whom three were asymptomatic, three suffered from isolated asthenia, and one was symptomatic with jaundice, abdominal pain, vomiting, diarrhea, malaise, and an altered general state. The symptomatic case died (Table 1). No additional case was identified despite an active search based on the interview of other general practitioners of the village and local laboratories. Samples collected during weeks 10 and 18 were systematically tested when patients gave their oral consent. Blood samples were taken from five blood donors living in village X who all tested negative for HEV RNA and anti-HEV IgM/IgG.

The hamlet with ten inhabitants had six houses, of which two were vacant. The seven cases lived in four different homes, three of which (home no. 2, 3 and 4, Table 1) were in the same small hamlet Y of village X. The fourth home (home no. 1, where the dead case lived) was outside hamlet Y, but the patient regularly visited his family (home no. 3).

The incubation period and the date of symptoms onset or the sampling date helped to deduce that the seven cases were exposed to the virus from week 2 of 2015 to week 12 of 2015. One case (case no. 3) was present in the village for only 2 weeks (weeks 07–2015 and 08–2015), which prompted us to investigate what happened during those two weeks.

The risk factors for HEV infection were investigated for six of the seven cases (Table 2). None had traveled outside France, but four of them had been in contact with domestic animals and three with farm animals. No one had been in direct contact with wild boar or deer.

We identified no common meal or common food consumption that could be a common source of contamination, and only two cases had eaten boar and deer. They had all eaten pork products, but these were purchased from different nation-wide hypermarkets. They had all consumed water from the public network.

### 3.2. Clinical Laboratory Investigations

**Figure 1:**
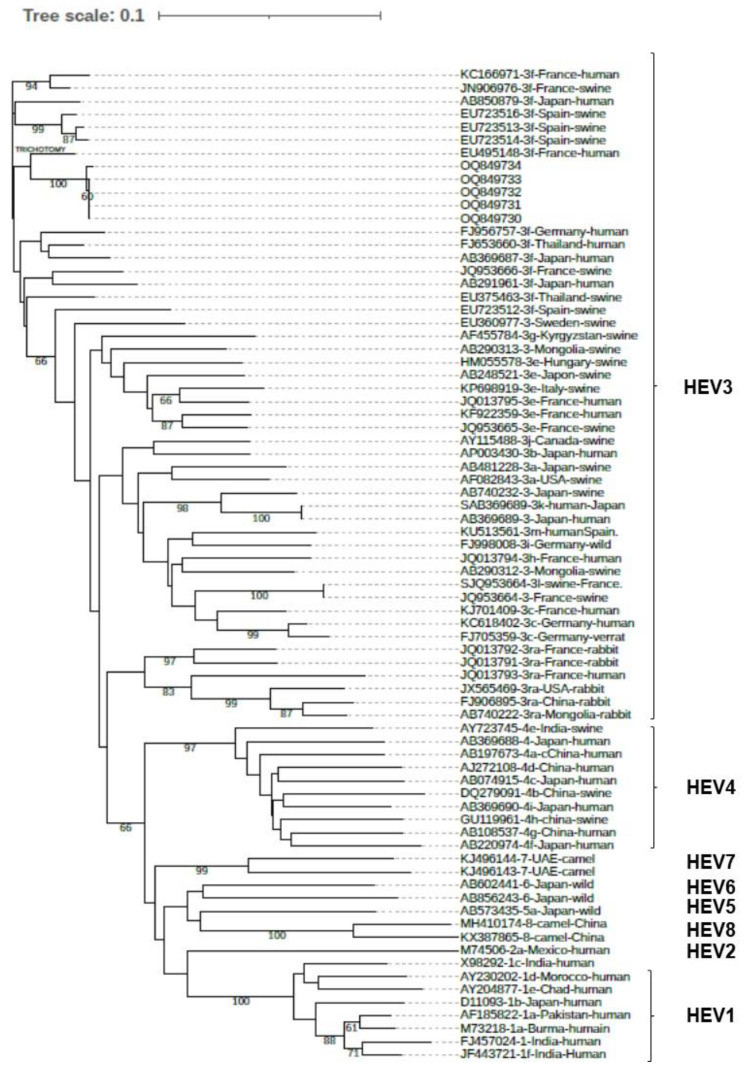
Phylogenetic tree based on partial ORF2 sequences (348 nt). Genbank accession numbers: OQ849730-OQ849734.

Six of the seven patients that were tested for anti-HEV IgM and IgG were positive at the time of sampling (Table 3). The sample from patient 1 was too small to perform both serologic and molecular analyses; only molecular analysis was performed. The other six patients except for patient 4 (5/6; 83.3%) were positive for HEV RNA (median HEV RNA concentration below 75 UI/mL (range: <75 IU/mL)). HEV subgenotype determination was possible for five samples and all were HEV3f (Figure 1). The subgenotype was confirmed by using blast NCBI tool. The virus samples from all five patients clustered together, with high bootstraps values, suggesting a common source of contamination. The sequences obtained for HEV isolated in patients 6 and 7 were shorter (about 250 nt). However, when comparing the nt sequence identities among the five different strains, identities were all >98.2%.

### 3.3. Environmental Investigations

Hamlet Y had four principal residences (including the three homes with cases) and two secondary residences (the owners were absent during the exposure period).

**Figure 2:**
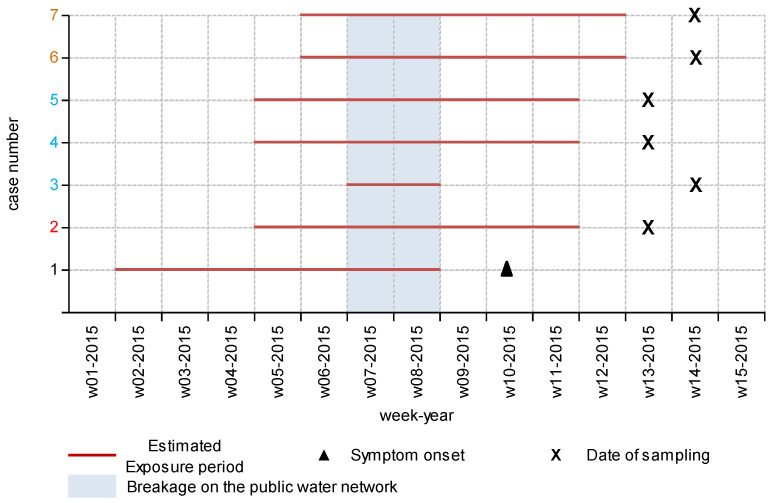
Estimated exposure period and symptom onset (or sampling date for the asymptomatic) for the seven cases of hepatitis E virus infection. Case number with the same color indicates co-inhabitants.

The public water network consisted of one principal network suppling the center of village X and a secondary network suppling hamlet Y. The secondary public network develop a leak during weeks 7 of 2015 and 8 of 2015, and the water became turbid. This incident occurred at the same time as the common exposure period of the seven cases (Figure 2). The first case occurred during week 10, three additional cases were diagnosed at week 13, and the last three cases were diagnosed one week later (week 14). The hamlet inhabitants reported significant turbidity (cloudy water) in their water network during February.

A private water network connected to a well was discovered in the house of a hamlet Y inhabitant, which was usually used to feed farm outbuildings and the inhabitant’s house. At the time of investigations and even before, there were no animals. This private network had a connection from the public water network but had no non-return element. In addition, the water catchment area of the private source was not protected to prevent cattle grazing.

Two water samples from the private network contained HEV RNA (Ct values: 35.37 and 36.38 in the house tap water and 31.70 and 31.67 in the well water). The genotype of the virus found in water was not determined because sequencing amplification failed due to the low HEV RNA concentration. The external amplification control (IVT RNA) revealed high PCR inhibition rates of 84,67% and 84,63% for the house tap water and the well water, respectively (in pure RNA extracts).

## 4. Discussion

Following the report of cases of HEV infection by a general practitioner, investigations were performed to confirm the clustering of these cases and to identify the source of contamination.

The investigations confirmed the spatial and temporal proximity of the cases, with six of the seven living in the same hamlet. The seventh case regularly visited their family in the hamlet. Phylogenetic analysis confirmed that all the patients for whom HEV RNA sequence was determined were contaminated by the same HEV3f strain. The fact that all the sequences were not 100% identical could be explained by intra-host mutations. No additional case was identified despite an active search.

As all the patients were contaminated by the same strain, we looked for a common source of the virus. There was no common source in their food; thus, investigations focused on the water because all the patients drank water from the same public network. Although the HEV incubation period can vary from 15 to 60 days, one case stayed in the hamlet for only 14 days, which shortened the exposure period to 2 weeks. There was a fracture of the hamlet water supply during this period, making the water very turbid. This did not prevent all seven patients from drinking this water. It is very likely that the seven cases were infected via this contaminated water. Unfortunately, no water samples were collected at that time.

HEV1 and HEV2 are known to cause waterborne outbreaks in developing countries [20,21].

For instance, one of the largest HEV epidemics occurred in Kanpur (population, 2.1 million), India, in 1991. The incidence of icteric hepatitis from December 1990 to April 1991 among the inhabitants of 420 randomly sampled houses in 7 of the city’s 50 wards was 3.76% (138 out of 3666 individuals). Consequently, it was estimated that 79,091 persons in the city were affected [21]. An interesting study based on the surveillance of HEV in sewage and drinking water in a resettlement colony of Delhi was performed. A total of 141 cases of viral hepatitis were included during a 3-year period. Out of the 141 cases of viral hepatitis, 41 (29.08%) had evidence of HEV infection, based on the detection of IgM anti-HEV and/or RT-PCR targeting the region encoding ORF1. Sewage and drinking water samples were collected individually from the houses of all viral hepatitis patients, and a drinking water sample was collected daily from the main outlet of the resettlement colony of Gokulpuri. After analysis, 6 of the 141 sewage samples (4.25%) and 2 of the 141 drinking water samples (1.42%) were positive for HEV RNA. All the strains were HEV1, indicating fecal contamination of drinking water and thus supporting the feco-oral mode of transmission of the disease. In this study, the attack rate of hepatitis E virus infection was found to be 0.59 per thousand individuals per year in the population. In previous outbreaks, the attack rates of viral hepatitis E have varied between 0.19 per thousand and 1.7 per thousand [20]. HEV3 has also been detected in shellfish from coastal waters in Europe, and thus, shellfish consumption is another possible route for HEV transmission [22,23]. This is reminiscent of hepatitis A virus (HAV) infection, which is linked to shellfish consumption, causing a debilitating disease and, occasionally, death [24,25,26]. HEV RNA was also detected in river water in the Netherlands [27], in surface water in Slovenia [28], and in sewage water in Europe [29,30]. However, the role of drinking water in spreading HEV is still poorly documented. A recent meta-analysis found HEV RNA in 4.7% (95% CI 0.0–15.8) of drinking waters tested in published studies [9].

The infectious dose for HEV is still not known. Studies on Cynomolgus monkeys indicated that oral infection needs at least 10^4.5^ particles [31]. A Swedish study found low concentrations of HEV RNA (10–130 IU/mL) in tap water [32]. Other naked viruses, such as enterovirus and adenovirus, remain viable in tap water [33]. A more recent Portuguese study found that 27.7% of the drinking water tested contained HEV infectious particles. Infectiousness of the particles was shown in vitro using Vero E6 cells [34]. The Vero E6 cultures were inoculated with 0.5 mL of concentrated (40×, in average) drinking water samples, equivalent to approximately 17.5 L of sampled water (1400 L, in average); humans usually drink 2 L per day.

Lastly, it remains to be determined how the HEV gets into the water network. The two main possibilities are: direct contamination at the break or contamination by a private source. Direct contamination at the break could be due to infiltration of contaminated surface water. In a jaundice outbreak in India, which was probably caused by HEV, the virus might have entered a drinking water pipeline that was damaged during sewer construction [35]. Another outbreak in India was explained by the close vicinity of the drinking water pipelines and a drainpipe. The drain water contaminated the domestic water supply [36]. Changing faulty water pipes and segregating them from drainpipes reduced the outbreak. While no HEV RNA was found in the public water supply at the time of our investigation, the samples were taken long after the break; HEV could have been cleared once the pipe had been repaired and flushed with chlorine. However, the effectiveness of chlorination on HEV remains to be determined [37]. HEV RNA was still detected for 7 days when HEV3 had been exposed to sodium hypochlorite and then inoculated onto PLC/PRF/5 cells, while viral RNA was detected for up to 33 days in cultures inoculated with untreated HEV [38]. These results need to be confirmed but suggest that HEV is at least somewhat sensitive to chlorination.

Another possibility is that the public network was contaminated by a private source. The break in the public network could have led to water from a private source entering the public water network due to the drop in pressure upstream. This is why such installation is forbidden in France. In addition, there was no anti-return device installed to prevent suction of the water from the well. The water sampled (source and tap) in the private network contained the HEV virus genome. However, as we were unable to compare the clinical and environmental strains, it is impossible to confirm this hypothesis. This private source serves only one house, whose owners showed no sign of HEV infection but refused to be tested. It may have been contaminated by the large population of wild boar living in this area [39,40]. Some studies indicate that surface water can be contaminated in the vicinity of pig production facilities, particularly if pig slurry is spread [28,41].

The fact that the owners do not present signs of HEV infection can be explained by the fact that most HEV infections are asymptomatic. During an outbreak of hepatitis E associated with a spit-roasted piglet occurring in France in 2013, 17 cases were identified, but 12 (71%) of the cases were asymptomatic. The diagnosis was based on the detection of biological markers (anti-HEV IgM) [7]. Since the owners were not tested, an HEV infection cannot be excluded based only on the absence of symptoms. Alternatively, the owners were already infected in the past by HEV and have protective anti-HEV IgG. Even though not precisely determined, a vaccine study suggests that the minimum protective concentration of antibodies is 2.5 World Health Organization (WHO) units/mL [42,43].

The main limit of this investigation is that we were unable to obtain the sequences of the HEV detected in the water due to the low HEV RNA concentration and the presence of PRC inhibitors. For low HEV RNA concentrations, the sequencing amplification works in 50% of attempts. In the absence of a HEV sequence from the environmental isolates, a case-control study would have been necessary to definitively confirm that residence in hamlet Y is associated with acquiring HEV infection.

## 5. Conclusions

To conclude, temporal and spatial investigations suggest that the private water supply containing HEV RNA was the probable source of the outbreak following the break in the network. Private water supplies still connected from the public network are frequent in rural areas, where they are a major contributor to public water pollution. Consequently, an environmental route of transmission should not be overlooked in rural areas.

## Figures and Tables

**Table 1:** Clinical and demographic characteristics of the seven cases of hepatitis E virus infection.

Case	Age (Years)	Sex	House Number Where the Patients Live	Sampling Date (Year-Week)	Comorbidities	Clinical Signs	Disease Outcome	Estimated Exposure Period (Week)
Case 1	78	Male	1	2015-10	Diabetes, heart failure, chronic alcoholism	Jaundice, vomiting, diarrhea, alteration of the general state	Death	2–8
Case 2	46	Female	2	2015-13	-	Asthenia	Self-limiting	5–11
Case 3	63	Female	3	2015-14	-	Asthenia	Self-limiting	7–8
Case 4	60	Male	3	2015-13	-	None	-	5–11
Case 5	27	Male	3	2015-13	-	Asthenia	Self-limiting	5–11
Case 6	58	Male	4	2015-14	-	None	-	6–12
Case 7	62	Male	4	2015-14	-	None	-	6–12

**Table 2:** Risk exposures for six cases of hepatitis E virus infection.

Risk Factor	Number of Patients
Travel abroad	0/6
Contact with:	
Domestic animals	4/6
Farm animals	3/6
Boar or deer	0/6
Consumption of pork products:	
Cured ham	6/6
Bacon	6/6
Dry-cured sausage	6/6
Garlic sausage	5/6
Sausage	5/6
Pork roast	5/6
Boiled ham	4/6
Liver pâté	4/6
Black pudding	1/6
Raw pork liver products	0/6
Consumption of shellfish	2/6
Consumption of water from public network	6/6

**Table 3:** Clinical laboratory results for the seven cases of hepatitis E virus infection.

Case	Detection of Anti-HEV IgM	Detection of HEV RNA	HEV RNA Concentration (IU/mL)	Genotype/Subgenotype
1	not tested	positive	96,750	3f
2	positive	positive	<75	3f
3	positive	positive	325	-
4	positive	negative	-	-
5	positive	positive	<75	3f
6	positive	positive	<75	3f
7	positive	positive	<75	3f

## Data Availability

Sequences were deposited into GenBank: OQ849730-OQ849734.
